# Evaluation of computer-assisted mandibular reconstruction with vascularized iliac crest bone graft compared to conventional surgery: a randomized prospective clinical trial

**DOI:** 10.1186/1745-6215-15-114

**Published:** 2014-04-09

**Authors:** Nassim Ayoub, Alireza Ghassemi, Majeed Rana, Marcus Gerressen, Dieter Riediger, Frank Hölzle, Ali Modabber

**Affiliations:** 1 Department of Oral, Maxillofacial and Plastic Facial Surgery, University Hospital of the RWTH Aachen, Pauwelsstraße 30, 52074 Aachen, Germany; 2 Department of Oral and Maxillofacial Surgery, Hannover Medical School, Carl-Neuberg-Straße 1, 30625 Hannover, Germany

**Keywords:** Computer-assisted surgery, Mandibular reconstruction, Vascularized iliac crest bone graft, Surgical guide, Virtual planning

## Abstract

**Background:**

Computer-assisted surgery plays an increasingly important role in mandibular reconstruction, ensuring the best possible masticatory function and aesthetic outcome.

**Methods:**

Twenty patients were randomly assigned to computer-assisted or conventional mandibular reconstruction with vascularized iliac crest bone graft in a prospective study design.

Virtual surgical planning was based on preoperative CT-data using specific surgical planning software. A rapid prototyping guide transferred the virtual surgery plan to the operation site. During surgery the transplant ischemic time, reconstruction time, time for shaping the transplant and amount of bone removed were measured. Additionally, the difference in the intercondylar distance before and after surgery was calculated.

**Results:**

Computer-assisted surgery shortened the time of transplant ischemia (*P* < 0.005) and defect reconstruction (*P* < 0.001) compared to conventional surgery. The time to saw and shape the transplant at the donor site was shorter using conventional surgery (*P* < 0.005); therefore, the overall time for surgery didn’t change (*P* = 0.527). In the computer-assisted group, the amount of bone harvested equaled the defect size, whereas the transplant size in the conventional group exceeded the defect site by 16.8 ± 5.6 mm (*P* < 0.001) on average. The intercondylar distance before compared to after surgery was less affected in the computer-assisted than in the conventional group (*P* < 0.001).

**Conclusions:**

The presented study shows that computer-assisted surgery can help reduce the time for mandibular defect reconstruction and consequently the transplant ischemic time. In the computer-assisted group, the iliac crest donor site defect was downsized and the postoperative condyle position was less altered, reducing possible risks of postoperative complications and donor site morbidity.

**Trial Registration:**

DRKS00005181.

## Background

In the field of oral and maxillofacial surgery, surgeons are often confronted with the need for complex reconstructions of bony mandibular defects. The reasons for these defects range from severe osteomyelitis to benign and malign mandibular tumors to accidents.

The aim of mandibular reconstruction is to achieve the best possible functional and aesthetic outcome; therefore, bone grafts play an important role.

The use of iliac crest bone grafts for mandibular reconstruction started in the early twentieth century [1]. With the introduction of microvascular surgery, bony reconstruction has been revolutionized because of the possibility of vascularized bone grafts. Microsurgically revascularized bone grafts lead to higher graft survival rates and improved functional and aesthetic outcomes [2-4].

There are several different opinions about the best possible bone graft to use. The most common bone grafts used are the fibula and iliac crest graft. Other options are the scapula or radius graft. On the one hand, the selection depends on the defect size, location, need for soft tissue and status of the recipient vessels; on the other hand, the availability of donor sites is also important [3].

In defects where no additional tissue is needed, the microsurgically revascularized iliac crest bone graft has some advantages compared to other bone grafts, because of its large amount of bone, rich cancellous blood supply and compact cortex. This makes it ideal for plate fixation and endosseous implants for dental rehabilitation [5]. The higher donor site morbidity of iliac crest bone grafts stated by others has not been confirmed in recent studies [6].

Nowadays computer-assisted surgery is becoming more and more important in the field of oral and maxillofacial surgery, because of the possibility of simulating almost every scenario imaginable with a high accuracy [7-9]. In the past, the success rate of a graft and the aesthetic outcome depended only on the surgeon’s competence and experience. Today, with the help of preoperatively taken digital CT-data it is possible to generate virtual 3D models of the facial skeleton, illustrating the defect. CT-data on the donor region gives precise information on the possible shape, size and placement of the bone graft needed [10]. With the help of angiographic CT-data it is even possible to determine the exact location of the nourishing vessels [11], thereby decreasing the risk of transplant failure. Previous studies have also shown a reduction in operating time and a significant decrease in complications [12,13]. Because of the high accuracy of computer-assisted surgery, better aesthetic and functional outcomes have been declared [14,15].

Nowadays different variations of computer-assisted surgery are used. The most popular is the production of a stereolithographic three-dimensional replica of the mandible and a transplant cutting guide, which is applied at the transplant donor site [16]. Other methods use prebent plates whose curvature is calculated preoperatively on the virtual three-dimensional model [17].

Another method described by Hou and colleagues is the use of a pre-shaped titanium mesh implant, which surrounds a vascularized fibular osteomyocutaneous flap [18].

Because most studies using computer-assisted surgery through a transplant cutting guide concentrate on the fibula as the donor site, further studies about computer-assisted mandibular reconstruction with iliac crest bone grafts are absent.

The aim of this study was to evaluate the benefits of computer-assisted mandibular reconstruction with iliac crest bone grafts regarding the intraoperative time for transplant shaping, ischemia, duration of surgery, amount of bone removed and the change in postoperative condyle position compared to conventional surgery.

## Patients and Methods

The study was approved by the local ethics committee at Aachen University, Germany (EK 163/11).

After institutional approval and written informed consent were obtained, 20 patients were randomly divided into two equal groups using the computer program RandList® (DatInf GmbH, Tübingen, Germany). The sample size of ten patients per group is based on a 5 % level of significance and a power of 80 % using previous data of a clinical pilot study [19], with the result of a minimum sample size of seven patients per group. One group received conventional surgical treatment, whereas the second group underwent computer-assisted surgical planning consisting of individual surgical guides. The patients’ characteristics and diagnoses are shown in Table 1. All patients received mandibular reconstruction with free vascularized iliac crest bone grafts. Times for harvesting and shaping the transplant at the donor site, shaping of the transplant at the defect site, reconstruction, osteosynthesis, ischemia and overall operation time were measured during surgery.

**Table 1:** Patients characteristics, diagnosis, surgical treatment, type of reconstruction, anastomosis

**Case number**	**Age/Gender**	**Diagnosis**	**Defect size**	**Location**	**Surgical treatment**	**Number of osteotomies**	**Type of reconstruction**	**Arterial anastomosis**	**Venous anastomosis**
1	33/f	Ameloblastoma	90.2 mm		Computer-assisted	2	Secondary	A. carotis externa	V. thyroidea superior
2	36/f	Osteomyelitis	94.8 mm		Computer-assisted	2	Primary	A. facialis	V. facialis
3	58/m	Bisphosphonate-related osteonecrosis of the jaw	92.8 mm		Computer-assisted	2	Primary	A. thyroidea superior	V. retromandibularis
4	46/f	Osteomyelitis	66.6 mm		Computer-assisted	3	Secondary	A. lingualis	V. jugularis externa
5	22/m	Ewing sarcoma	118.6 mm		Computer-assisted	2	Primary	A. lingualis	V. jugularis interna
6	66/m	Squamous cell carcinoma	107.0 mm		Computer-assisted	5	Secondary	A. thyroidea superior	V. jugularis interna
7	44/f	Ameloblastoma	82.8 mm		Computer-assisted	2	Primary	A. facialis	V. facialis
8	81/f	Ameloblastoma	72.8 mm		Computer-assisted	3	Secondary	A. lingualis	V. jugularis interna
9	75/f	Squamous cell carcinoma	80.1 mm		Computer-assisted	2	Secondary	A. carotis externa	V. jugularis anterior
10	62/m	Squamous cell carcinoma	70.5 mm		Computer-assisted	2	Secondary	A. carotis externa	V. retromandibularis
11	43/m	Osteomyelitis	109.5 mm		Conventional treatment	2	Secondary	A. thyroidea superior	V. facialis
12	41/m	Osteomyelitis	103.0 mm		Conventional treatment	3	Secondary	A. thyroidea superior	V. thyroidea superior
13	29/f	Keratocyst	82.8 mm		Conventional treatment	2	Primary	A. thyroidea superior	V. thyroidea superior
14	45/m	Squamous cell carcinoma	106.0 mm		Conventional treatment	4	Secondary	A. carotis externa	V. facialis
15	61/m	Osteoradionecrosis	62.9 mm		Conventional treatment	2	Primary	A. lingualis	V. jugularis externa
16	68/f	Ameloblastoma	74.2 mm		Conventional treatment	2	Secondary	A. facialis	V. facialis
17	69/f	Osteomyelitis	63.0 mm		Conventional treatment	2	Secondary	A. lingualis	V. jugularis externa
18	59/m	Bisphosphonate-related osteonecrosis of the jaw	67.7 mm		Conventional treatment	2	Primary	A. thyroidea superior	V. jugularis interna
19	71/w	Osteomyelitis	67.7 mm		Conventional treatment	2	Secondary	A. thyroidea superior	V. jugularis externa
20	61/m	Squamous cell carcinoma	96.1 mm		Conventional treatment	3	Secondary	A. thyroidea superior	V. jugularis interna

The shaping time at the donor site is the time needed to saw and shape the transplant at the donor site before transection of the nourishing vessels. The time for shaping at the defect site is defined as the time to customize the transplant at the defect site after the dissection of the pedicle. Osteosynthesis time is the time needed to fixate the transplant in the defect site by osteosynthesis. So reconstruction time includes the time to shape the transplant and perform the osteosynthesis at the defect site, whereas ischemia is defined as the time from dissection of the pedicle until perfusion of the transplant is restored.

Additionally, the size of the harvested bone was measured and compared to the amount of bone needed to fill the defect site.

ICU time, period of postoperative hospitalization and units of erythrocyte concentrate (1 unit equals 0.5 liter of erythrocyte concentrate) were also taken into account.

**Figure 1:**
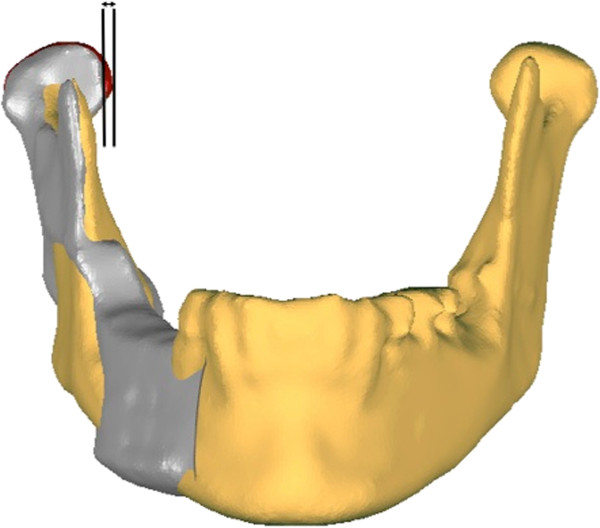
**The absolute deviation in the pre- and postoperative intercondylar distance was measured by superimposition of the preoperative with the postoperative three-dimensional model of the mandible.** Grey indicates the iliac crest transplant and the altered postoperative position of the mandible with the condyle. The preoperative position of the condyle is marked in red.

Moreover, the difference in condyle position before and after surgery was evaluated. To compare the pre- and postoperative condyle position, the intercondylar distance was measured using three-dimensional models of the mandible before and after surgery. The models were imported into the Geomagic Studio software (Geomagic, Morrisville, NC, USA) using the STL-format. One side of the preoperative mandible including the condyle was matched with the postoperative reconstructed mandible to measure the absolute deviation in the intercondylar distance (Figure 1).

### Statistical analysis

For statistical analysis the Student *t*-test was used. The level of significance was set at *P* ≤ 0.05. The *χ*^2^-test with a level of significance at *P* ≤ 0.05 was used for analyzing gender. All data are expressed as mean values ± standard deviation. Statistical calculations were performed under SPSS v14 (SPSS, Chicago, IL, USA) running on a Windows 7 computer (Microsoft Corporation, Redmond, WA, USA).

### Surgical planning and rapid prototyping

Virtual surgical planning was carried out in ten patients. As previously described [14,15], we used the commercially available software ProPlan CMF (Materialise NV, Leuven, Belgium).

**Figure 2:**
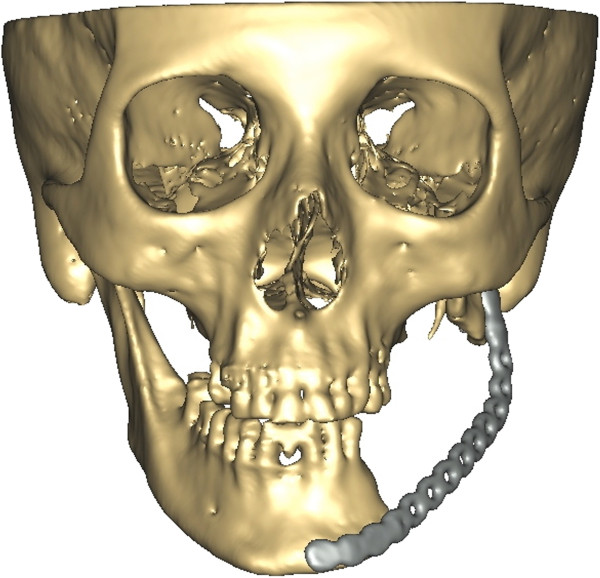
Preoperative three-dimensional model of the facial skeleton.

Preoperative CT-data in DICOM format was imported into the planning software ProPlan CMF. In the next step, a virtual three-dimensional model of the facial skeleton and iliac crest was segmented, thereby removing all artifacts. The result was a high quality three-dimensional visualization (Figure 2). In cases of secondary reconstruction, the healthy side of the mandible was mirrored on the site of the defect or previous CT-data was used, whereas in primary reconstruction the shape of the mandible before resection was set as a reference for reconstruction.

**Figure 3:**
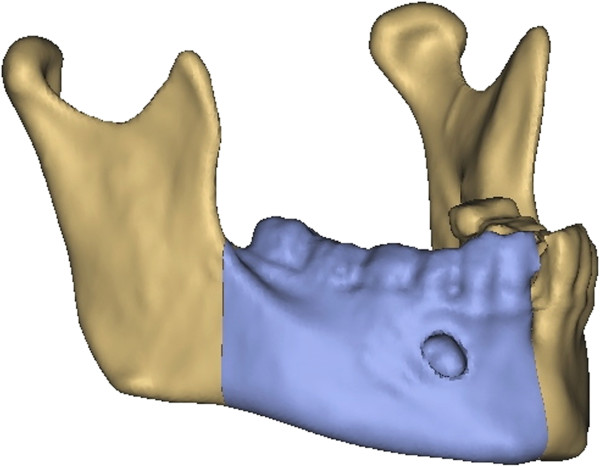
Virtual resection of the right mandible in a case of primary reconstruction.

**Figure 4:**
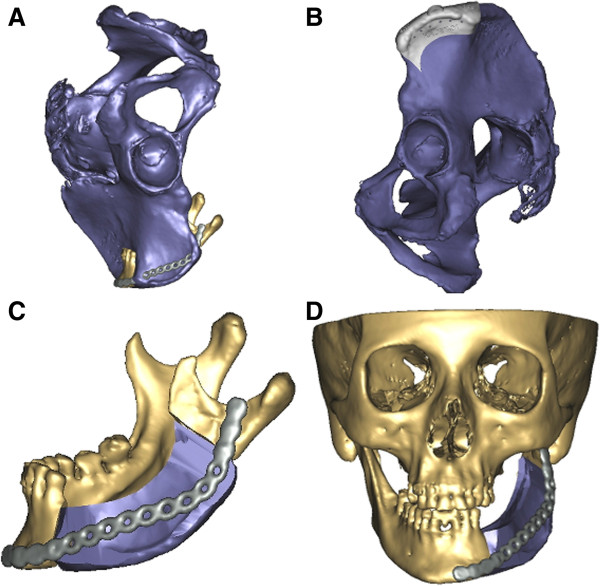
**Preoperative virtual planning. (A)** Iliac crest is moved into the defect site of the left mandible. **(B)** Transplant cutting guide placed on the left iliac crest. **(C)** Virtually-cut iliac crest graft placed at the defect site of the mandible. **(D)** Whole facial skeleton with the final iliac crest graft in position.

**Figure 5:**
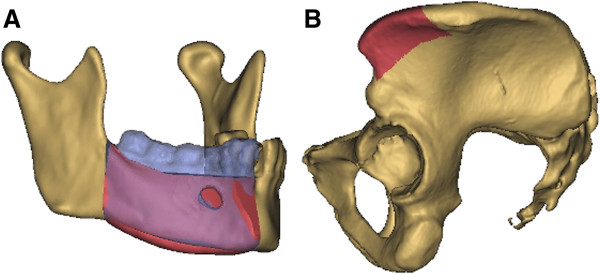
**Primary reconstruction of the mandible. (A)** For primary reconstruction the resected part of the right mandible (blue) acted as reference for the left iliac crest graft (red). **(B)** Iliac crest graft (red) marked on the pelvis.

In primary reconstruction, a virtual resection of the afflicted part of the mandible was carried out regarding the preoperative CT-data (Figure 3). Using different surgical tools available in the software such as osteotomy modus, mirroring tool and reconstruction wizard a reconstruction design could be developed, whereby the nourishing vessels of the iliac crest bone graft were taken into account (Figure 4). In consultation with the surgeon, different reconstruction plans were discussed according to the best possible curvature, graft position, jawbone distance and condyle position. Most important were the occlusion regarding possible dental rehabilitation and the aesthetic outcome (Figure 5).

**Figure 6:**
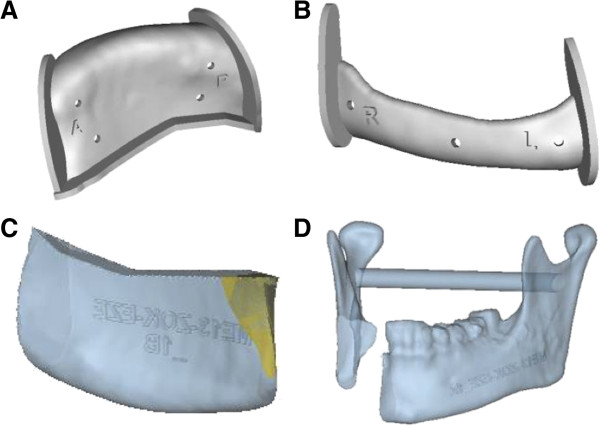
**Guides and models.** The transplant cutting guide **(A)** and resection guide of the mandible **(B)** are manufactured via a rapid prototyping selective laser sintering method. Stereolithographic model of the virtually-created transplant of the iliac crest **(C)** and the mandible after resection **(D)**.

After fine-tuning, the generated three-dimensional transplant and models were imported into 3matic-Software (Materialise NV, Leuven, Belgium) using the STL-format. In this software, a uniquely fitting transplant cutting guide was designed, which was manufactured via a rapid prototyping selective laser sintering method (Figure 6a).

All necessary information in the virtual plan, including the dimensions, resection sites, and position and angulation of osteotomies, were included in the produced transplant cutting guide.

In cases of primary reconstruction, an additional resection guide for the mandible was produced to ensure the exact preoperatively planned resection of the afflicted part of the mandible (Figure 6b).

Using a stereolithographic technique, a skull model with the defect and a model of the virtually-shaped transplant, which functioned as an intraoperative back up for graft shaping, was manufactured (Figure 6 cd).

### Surgical procedure

In each group a two-team approach was used in which one team prepared the defect region, vessels and, in cases of primary reconstruction, resected the afflicted part of the mandible, while the second team elevated the iliac crest bone graft. The teams always consisted of the same surgeons.

**Figure 7:**
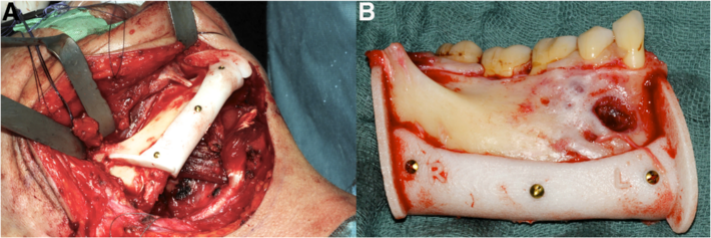
**Resection of the mandible. (A)** Fixed resection guide on the mandible using mini screws. **(B)** Resected portion of the mandible with resection guide.

**Figure 8:**
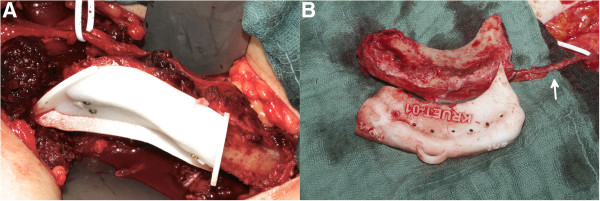
**Harvest of the iliac crest transplant. (A)** Transplant cutting guide temporarily fixed on the iliac crest. **(B)** Still-pedicled transplant with cutting guide. The arrow marks the pedicle.

**Figure 9:**
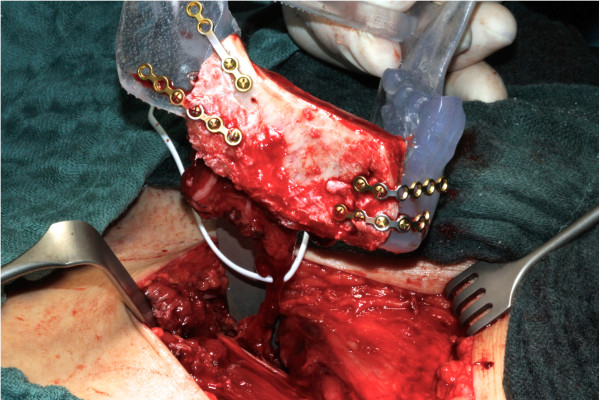
Using the stereolithographic model to pre-bend mini plates on the still-pedicled iliac crest graft.

In the computer-assisted surgical planning group, the surgeon was provided with the transplant cutting guide, transplant model, defect model and resection guide in cases of primary reconstruction (Figure 7). After preparing the iliac crest bone graft, the transplant cutting guide was fixed temporarily to the lateral side of the iliac crest using osteosynthesis screws. In the next step, the graft was sawn according to the transplant cutting guide, taking care not to injure the vessels (Figure 8). In cases of planned osteotomies, the transplant cutting guide had an additional sawing slot. Using the defect model it was also possible to partially bend and fix the osteosynthesis plates on the transplant before transection of the pedicle (Figure 9).

**Figure 10:**
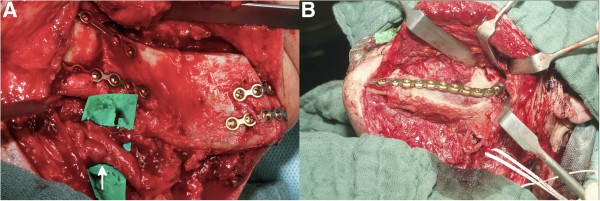
**Primary and secondary reconstruction of the mandible. (A)** Iliac crest transplant fixed at the right mandible in a case of primary reconstruction. Arrow indicates the anastomosis. **(B)** Iliac crest transplant in a case of secondary reconstruction placed at the defect site of the left mandible.

Following transection of the pedicle the transplant, if necessary, was further shaped at the defect site and afterwards immediately inserted into the defect and fixed using the previously applied osteosynthesis plates (Figure 10).

In the conventional surgical treatment group, the surgeon osteotomized the iliac crest according to the defect size, which was measured by the team preparing the defect region. Afterwards, the vessels were transected and the graft was shaped for a better fit at the defect site before applying the osteosynthesis plates and conducting anastomosis.

## Results

The patients` mean age who received computer-assisted surgery was 52.3 (range 22 to 81) years and 54.7 (range 29 to 71) years for conventional reconstruction. There were no significant differences concerning age, gender, defect size, osteotomies of the grafts, duration of postoperative hospitalization, duration in the ICU or applied units of erythrocyte concentrates between the groups (Table 2).

**Table 2:** Baseline characteristics of patients

	**Computer-assisted**	**Conventional**	***P*-value**
Female gender (n/total (%)	6/10 (60)	4/10 (40)	0.371
Age (years)	52.3 ± 19.2	54.7 ± 14.2	0.756
Defect size (mm)	87.6 ± 16.5	83.3 ± 18.7	0.590
Postoperative hospitalization duration (days)	17.5 ± 7.4	19.1 ± 9.7	0.683
ICU duration (days)	2.0 ± 0.9	2.1 ± 2.1	0.894
Erythrocyte concentrate application (unit = 0.5 liter)	2.0 ± 1.8	2.2 ± 1.5	0.791
Number of osteotomies	2.5 ± 1.0	2.4 ± 0.7	0.795

**Figure 11:**
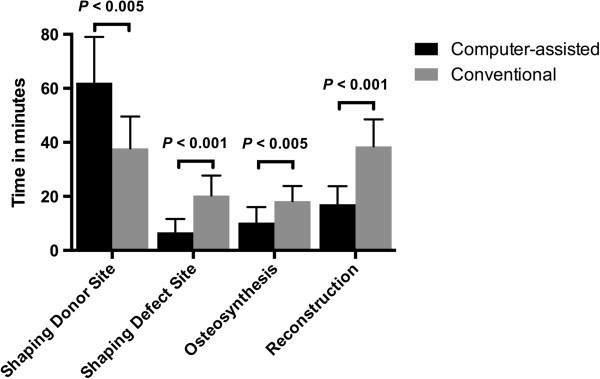
**Time (minutes ± standard deviation) was measured during surgery.** The time needed for shaping the transplant at the donor site was significantly shorter in the computer-assisted group, whereas the time for shaping the transplant at the defect site, osteosynthesis and reconstruction was reduced using computer-assisted surgery.

The time from shaping the transplant at the donor site until dissection of the pedicle took 62.1 ± 17.0 minutes in the computer-assisted group in contrast to 37.8 ± 11.8 minutes (*P* < 0.005) in the conventional group (Figure 11).

Figure 11 also shows the time spent shaping the transplant at the defect site, which was significantly lower in the computer-assisted group compared to the conventional group (6.2 ± 4.9 versus 20.3 ± 7.4 minutes; *P* < 0.001).

Additionally, the time for osteosynthesis of the transplant, shown in Figure 11, was significantly reduced in the computer-assisted group (10.1 ± 5.4 versus 18.2 ± 5.6 minutes; *P* < 0.005), thereby reducing the overall reconstruction time as well (16.4 ± 6.7 versus 38.5 ± 10.0 minutes; *P* < 0.001).

**Figure 12:**
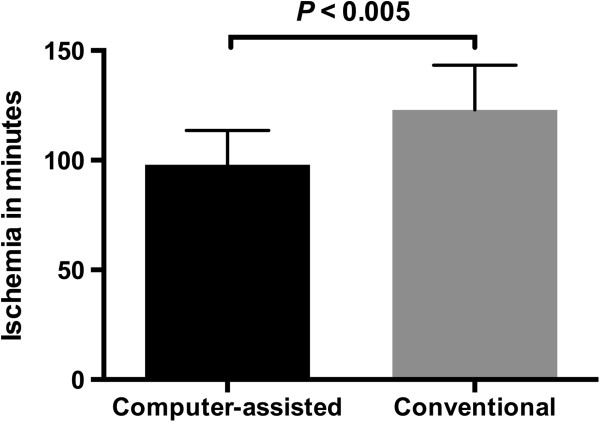
**Ischemic time (minutes ± standard deviation) of the iliac crest bone graft.** In the computer-assisted group the ischemia of the transplant was significantly less compared to conventional surgery.

**Figure 13:**
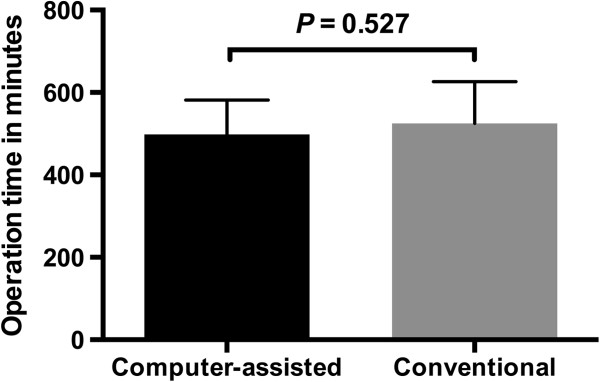
**Operation time (minutes ± standard deviation) comparing both groups.** No change in overall operation time was observed.

Furthermore, a decrease in the ischemic time in the computer-assisted group was observed (96.1 ± 15.8 versus 122.9 ± 20.4 minutes; *P* < 0.005) (Figure 12), whereas the overall operation time showed no significant difference between the two groups (498.5 ± 83.4 versus 525.2 ± 100.9 minutes; *P* = 0.527) (Figure 13).

**Figure 14:**
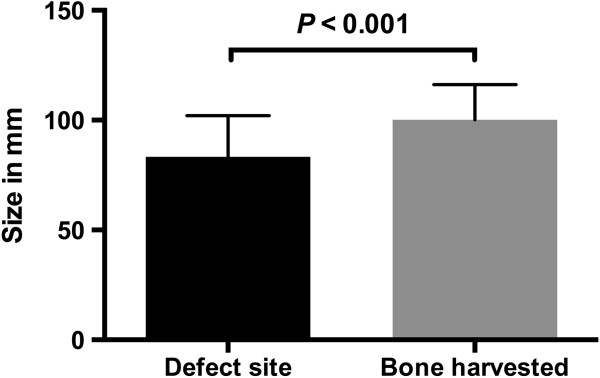
**Measured size (mm ± standard deviation) of the defect and of the bone harvested using conventional surgery.** In the conventional group, the amount of bone harvested was significantly higher compared to the defect size.

In the computer-assisted group, the amount of bone harvested from the iliac crest showed no significant difference regarding the defect size (87.6 ± 16.5 mm), while the average transplant size in the conventional group exceeded the defect size significantly by 16.8 mm (±5.6 mm; *P* < 0.001), shown in Figure 14.

**Figure 15:**
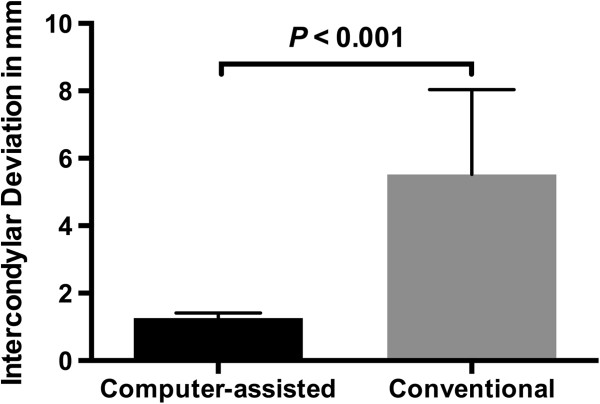
**Absolute preoperative to postoperative intercondylar deviation (mm ± standard deviation).** Using computer-assisted surgery the absolute intercondylar deviation comparing pre- and postoperative condyle position was significantly lower in contrast to conventional surgery.

Comparing the position of the condyle before and after surgery in both groups, using computer-assisted surgery a significantly lower discrepancy in the intercondylar distance was observed compared to conventional surgery (1.3 ± 0.2 versus 5.5 ± 2.5 mm; *P* < 0.001) (Figure 15).

Due to venous thrombosis, one transplant in each group failed, which led to a transplant survival rate of 90 % in both groups.

## Discussion

Bony reconstruction of the mandible can be achieved by different types of transplants derived from the fibula, iliac crest, scapula or radius.

To decide which transplant is best to use, the setting at the defect site is crucial. If the defect site lacks soft tissue most surgeons prefer an osteomyocutaneous fibular flap [20], because of the possibility of a tissue island. Furthermore, the recipient vessels have to be taken into account. Patients who have already undergone multiple surgeries or radiation may lack suitable vessels. In contrast to the iliac crest, the fibular flap has a relatively long pedicle, which allows conducting anastomosis even on the contralateral side of the defect. In cases of appropriate vessel situation and no required tissue, iliac crest bone grafts have several advantages including a compact cortex and large amount of bone, which has a positive influence on plate fixation and postoperative insertion of dental implants.

With the help of computer-assisted surgery, different virtual scenarios for mandibular reconstruction can be taken into consideration, which leads to a clinical benefit of predictable anatomical dimensions, reconstruction limitations and possible complications [21,22]. Thereby, computer-assisted surgery can be used for primary and secondary reconstruction. In cases of malignant tumors, secondary reconstruction is recommended, because it is not possible to extend the surgical plan intraoperatively [14]. If primary reconstruction is favored or inevitable it is possible to plan two different surgical approaches, thereby producing different surgical guides and models and expanding the flexibility of reconstruction.

A few studies have already tried to highlight the benefits of computer-assisted surgery, mainly concentrating on fibula flaps. Therefore, a randomized prospective study was needed, especially for iliac crest bone grafts.

We used a stereolithographic three-dimensional replica with a transplant cutting guide, which is the most common method of transferring the virtual planning to real time surgery.

Regarding the transplant survival rate, the ischemic time plays a crucial role. Keeping ischemic time as short as possible increases the transplant survival rate [23].

This study was able to show that with the help of computer-assisted surgery the ischemic time is reduced compared to conventional surgery. Ischemic time depends on several different elements, starting with the transection of the pedicle and ending with the restoration of perfusion. After transecting the pedicle, the transplant needs to be shaped at the defect site to ensure the best possible aesthetic and functional outcome. The time needed for shaping the transplant at the defect site increases ischemia. Therefore, the shaping procedure at the defect site should be as rapid as possible. Using computer-assisted surgery, the time for shaping the transplant at the defect site was decreased. Another important parameter that influences the length of ischemia is the time needed for osteosynthesis. Osteosynthesis should be carried out with great care because it affects the osseous integration of the graft and determines the graft position. Compared to conventional surgery, the time needed for osteosynthesis could be reduced using computer-assisted surgery, thereby reducing the overall ischemic time.

Another benefit of using computer-assisted surgery stated in other studies is a lower overall operation time [24]. However, our results did not confirm these findings. The overall operation time in this study showed no difference comparing both groups. This is due to the fact that shaping the transplant at the donor site took significantly more time in the computer-assisted group. Applying the transplant cutting guide at the iliac crest and shaping the transplant accordingly takes more time, but it has to be mentioned that while shaping the transplant at the donor site the transection of the pedicle has not been conducted yet; therefore, perfusion of the transplant is assured. It is even already possible to bend and partially apply the osteosynthesis plates using the three-dimensional replica provided, while the transplant is still pedicled. Therefore, computer-assisted surgery helps transfer time-consuming processes like transplant shaping, which usually take place during ischemia, to a point in the surgery where perfusion is still ongoing. Afterwards, the additional time required to shape the transplant at the donor site is saved at the defect site. It also must be taken into account that the nourishing vessels of the iliac crest bone graft are very fragile, therefore great care has to be taken while shaping the still-pedicled transplant.

Furthermore, postoperative donor site morbidity plays a critical role regarding patient satisfaction and quality of life. Ghassemi et al. [25] were able to show a correlation between the amount of bone harvested and postoperative complications; therefore, the harvested transplant should not contain unnecessary bone. With the help of computer-assisted surgery we were able to avoid harvesting bone unnecessarily, due to precise preoperative planning and uniquely fitting transplant cutting guide, which allows precise sawing and shaping of the transplant. In contrast to the computer-assisted group, the transplant size using conventional surgery exceeded the defect size on average by 16.8 mm, thereby increasing the risk of donor site morbidities.

Temporomandibular joint dysfunction can be associated with malocclusion, false condyle position and condylar disk displacement [26,27]. To avert postoperative temporomandibular joint dysfunction, alterations in the condyle position or malocclusion should be avoided; this improves patients’ postoperative satisfaction and quality of life. However, significantly smaller discrepancies in the position of the condyle were found in the computer-assisted group. On average, conventional surgery altered the intercondylar distance by 5.5 mm, compared to 1.3 mm in the computer-assisted group; therefore, the use of computer-assisted surgery can decrease the risk of postoperative temporomandibular joint dysfunction.

When discussing computer-assisted surgery, the higher costs have to be mentioned. Without any doubt, the costs of using computer-assisted surgery are higher compared to conventional surgery. The main factors causing the higher costs are the production of the transplant cutting guides, skull and transplant models. Studies have shown that the benefits listed above clearly outweigh the cost of computer-assisted surgery [24,28]. In our study, the overall operation time did not change significantly, therefore cost savings regarding a reduced operation time cannot be expected as a main benefit. Nonetheless, we were able to highlight certain significant advantages of computer-assisted surgery like a lower change in intercondylar distance, which could lead to less postoperative temporomandibular joint dysfunctions. Furthermore, our results show the reduction to a minimum of unnecessarily harvested iliac crest bone, which may reduce postoperative donor site morbidity and associated costs. In addition, we could show a significant drop in ischemic time. The ischemic time is considered as one of the main causes of transplant failure, therefore a decrease of the ischemic time could reduce the risks for transplant failure. In our opinion, these benefits of computer-assisted surgery outweigh the costs on the long run.

## Conclusion

This is the first randomized prospective study comparing computer-assisted mandibular reconstruction with conventional surgery using iliac crest bone grafts. The presented study shows that computer-assisted reconstruction reduces ischemic time and transplant-shaping time at the defect site as well as requiring a smaller amount of harvested bone than conventional surgery. Moreover, a significantly smaller alteration in condyle position could be shown in the computer-assisted group.

## Abbreviations

A: arteria; CT: computed tomography; DICOM: digital imaging and communications in medicine; f: female; ICU: intensive care unit; m: male; mm: millimeter; V: vena.

## Competing interests

The authors declare that they have no competing interests.

## Authors’ contributions

NA: data collection and analysis, manuscript writing, final approval of the manuscript. AG: data collection and interpretation, critical revision and final approval of the manuscript. MR: data collection and interpretation, critical revision and final approval of the manuscript. MG: data collection and interpretation, critical revision and final approval of the manuscript. DR: data collection and interpretation, critical revision and final approval of the manuscript. FH: data collection and interpretation, critical revision and final approval of the manuscript. AM: conception and design, data collection, analysis and interpretation, drafting, critical revision and final approval of the manuscript. All authors read and approved the final manuscript.
